# Rapid Sequential Spread of Two *Wolbachia* Variants in *Drosophila simulans*

**DOI:** 10.1371/journal.ppat.1003607

**Published:** 2013-09-12

**Authors:** Peter Kriesner, Ary A. Hoffmann, Siu F. Lee, Michael Turelli, Andrew R. Weeks

**Affiliations:** 1 Department of Genetics, University of Melbourne, Parkville, Victoria, Australia; 2 Department of Evolution and Ecology and Center for Population Biology, University of California, Davis, Davis, California, United States of America; CNRS - Université Lyon 1, France

## Abstract

The maternally inherited intracellular bacteria *Wolbachia* can manipulate host reproduction in various ways that foster frequency increases within and among host populations. Manipulations involving cytoplasmic incompatibility (CI), where matings between infected males and uninfected females produce non-viable embryos, are common in arthropods and produce a reproductive advantage for infected females. CI was associated with the spread of *Wolbachia* variant *w*Ri in Californian populations of *Drosophila simulans*, which was interpreted as a bistable wave, in which local infection frequencies tend to increase only once the infection becomes sufficiently common to offset imperfect maternal transmission and infection costs. However, maternally inherited *Wolbachia* are expected to evolve towards mutualism, and they are known to increase host fitness by protecting against infectious microbes or increasing fecundity. We describe the sequential spread over approximately 20 years in natural populations of *D. simulans* on the east coast of Australia of two *Wolbachia* variants (*w*Au and *w*Ri), only one of which causes significant CI, with *w*Ri displacing *w*Au since 2004. *Wolbachia* and mtDNA frequency data and analyses suggest that these dynamics, as well as the earlier spread in California, are best understood as Fisherian waves of favourable variants, in which local spread tends to occur from arbitrarily low frequencies. We discuss implications for *Wolbachia*-host dynamics and coevolution and for applications of *Wolbachia* to disease control.

## Author Summary

*Wolbachia* are bacteria that live within the cells of arthropod hosts and are widespread in many groups of insects. These bacteria can rapidly spread through a population through a process of cytoplasmic incompatibility whereby females uninfected by *Wolbachia* show embryo death when they mate with males carrying the bacteria. Because the infected females pass on *Wolbachia* to their offspring, this places them at a reproductive advantage, ensuring that the infection spreads through insect populations once it reaches a high enough frequency to overcome any negative fitness effects on its host. Yet while such a rapid spread has been predicted, it has rarely been observed in nature. Here we show that a *Wolbachia* infection of *Drosophila simulans* flies has spread very rapidly in eastern Australia, replacing another *Wolbachia* infection that has also spread in recent years. These invasions appear to have taken place from a very low frequency, implying that both infections are likely to have had a benefit to their hosts rather than a cost. These results have implications for the spread of *Wolbachia* infections currently being introduced into populations of mosquitoes and other insects for disease suppression.

## Introduction

The vertically-transmitted intracellular bacterium *Wolbachia* may be the most widespread [1]–[3] and evolutionarily significant endosymbiont [4], [5] of insects and other arthropods. *Wolbachia* induce many reproductive manipulations within hosts that increase their chance of spreading through females. Their ability to suppress other microbes in their hosts provides a novel method to control human vector-borne diseases such as dengue [6], [7] and malaria [8], [9]. Yet despite their ubiquity and potential importance in vector-borne disease control, there are few documented examples of *Wolbachia* infections spreading in natural host populations [10], [11].

The most commonly documented host reproductive manipulation by *Wolbachia* is cytoplasmic incompatibility (CI) [12], which reduces hatch rates for embryos produced when sperm from an infected male fertilizes uninfected ova or ova that carry an incompatible *Wolbachia* strain. CI provides a reproductive advantage to infected female hosts (whose infected eggs are protected from CI), and it can drive the spread of a *Wolbachia* infection within and among host populations [11], [13]–[15]. Several factors can affect the spread of CI-inducing *Wolbachia* infections, including the strength of incompatibility (quantified by *H*, the relative hatch rate of embryos from incompatible fertilizations), the maternal transmission frequency (1−*μ*, where *μ* is the frequency of uninfected ova produced by an infected female), and the fecundity of infected females relative to uninfected females (*F*, which can also approximate viability effects) [12].

Mathematical analyses [14]–[16] show that irrespective of the level of CI, as measured by *H* (0≤*H*≤1), if a CI-inducing infection satisfies *F*(1−*μ*)<1, it will tend to decrease in frequency when very rare. The reason is that the CI-induced reproductive advantage for infected females depends on the frequency of infected males, whereas disadvantages attributable to fitness costs, *F*<1, or imperfect maternal transmission, *μ*>0, are frequency independent. If the level of CI is sufficient that *F*(1−*μ*)>*H* (i.e., when the CI-inducing infection is very common, more infected offspring are produced by infected mothers than viable uninfected offspring are produced by uninfected mothers), the infection will tend to spread locally once it becomes sufficiently common. Hence for *Wolbachia* strains that satisfy *H*<*F*(1−*μ*)<1, we expect “bistable” dynamics [13]–[15], where there is an unstable equilibrium infection frequency, denoted *p*_u_, that satisfies 0<*p*_u_<1; *p*_u_ separates two stable equilibria, one at 0, the other a high frequency denoted *p*_s_ (which always exceeds ½, [17]). If maternal transmission is imperfect (*μ*>0), then *p*_s_<1 because uninfected individuals are continuously introduced. The unstable equilibrium frequency, *p*_u_, plays a central role in both local dynamics and spatial spread. Local dynamics depend on the initial infection frequency, denoted *p*_0_. If *p*_0_>*p*_u_, the infection frequency tends to increase towards *p*_s_; conversely if *p*_0_<*p*_u_, the frequency tends to decrease to 0.

Spatial dynamics are more complex, and predictions depend on the initial frequencies over an extended area. Nevertheless spatial spread from a localized introduction cannot occur if *p*_u_ is too large; roughly, bistable infections do not spread unless *p*_u_<½ (the exact condition is described in [13] and [18]). If a CI-inducing infection with *p*_u_<½ becomes established in a sufficiently large region (see Fig. 3 of [13]), it will tend to spread spatially at a rate determined by average dispersal distance of females and the intensity of CI. These bistable waves can be stopped by regional variation in population density or dispersal barriers [19].

In contrast to bistable infections, *Wolbachia* strains that provide a frequency-independent fitness advantage to infected hosts, such that *F*(1−*μ*)>1, would increase locally, even from low initial frequencies and regardless of whether they cause CI. Local spread would be followed by a “Fisherian” spatial wave [20], [21] that, unlike a bistable wave, is unlikely to be halted. For such infections, 0 is the unstable equilibrium and the unique stable equilibrium satisfies *p*_s_<1 if maternal transmission is imperfect [12]. As in [13], we use “Fisherian” to describe the spatial dynamics of variants that tend to increase even when locally very rare, unlike bistable variants that tend to increase only once they exceed a critical frequency *p*_u_>0. The mechanism that reduces the unstable equilibrium to zero may involve interactions between *Wolbachia*, *D. simulans* and various pathogenic microbes, as postulated by Fenton et al. [22]. Alternatively it may involve nutritional provisioning [23], [24] or other effects as yet unknown. In the absence of relevant field data, we approximate the effects of *Wolbachia* on *D. simulans* by a frequency-independent advantage that produces *F*(1−*μ*)>1. Nothing about our analyses or conclusions, which rest on the rapid sequential spread of two *Wolbachia* variants, requires a more detailed model. The key feature of Fisherian variants is that they spread much more rapidly than bistable variants, because small advance propagules can catalyze invasions of new areas [25, Ch. 5], in contrast to the steadily moving wave fronts expected with bistable dynamics. Unfortunately, the temporal and spatial resolutions of our data are insufficient for fitting mechanistic models of spatial spread.

Turelli & Hoffmann [11], [17] first documented a *Wolbachia* infection spreading within and among natural populations. A strain of *Wolbachia* (*w*Ri) was initially found infecting *Drosophila simulans* populations in southern California. Turelli and Hoffmann monitored the northward spread of *w*Ri over ten years (1985–1994). Because they repeatedly found both imperfect maternal transmission in nature and reduced relative fecundity for infected females in the laboratory [15], [17], they assumed *F*(1−*μ*)<1 and inferred bistable dynamics (i.e., *H*<*F*(1−*μ*)<1). Using field-based estimates of *μ*, *F* and *H*, their mathematical analyses implied *p*_s_≈0.94, in close agreement with relatively constant infection frequencies observed in several natural populations for over 20 years [26]. However, as emphasized by Jaenike [27] and elaborated by Carrington et al. [26], the CI-based prediction for *p*_s_ is quite insensitive to variation in the relative fecundity of infected females, *F*. Based on field estimates of *μ* near 0.045, *F*(1−*μ*)>1 requires only that infected females have a fecundity advantage on the order of 5%. We present new mtDNA data from recent California samples of *D. simulans* and from a 1961 southern California collection which suggest that *w*Ri invaded California less than 25 years before Hoffmann et al. [28] found it. This supports our new interpretation of Fisherian versus bistable spread in both California and Australia.

Other studies of *Wolbachia* in natural host populations have also assumed bistable dynamics [10], [29], in one case [29] based on observing very rare imperfect maternal transmission (*μ*≈0.014), in the other [10] based on an indication of spatial spread analogous to that seen in *D. simulans*. A definitive example of bistable dynamics comes from recent field releases of *Wolbachia*-infected *Aedes aegypti* mosquitoes. The *w*Mel *Wolbachia* infection was transferred in the laboratory from its native host *D. melanogaster* to *Ae. aegypti* as part of a novel strategy for blocking transmission of the human dengue virus [6], [7]. The observed rate of infection frequency increase within populations was consistent with significant fitness costs, and hence bistable dynamics; and low-frequency introductions into neighbouring populations have not led to *Wolbachia* establishment outside the release areas, as predicted with bistability (Eliminate Dengue Team, unpubl. data).

These mosquito data provide experimental support for bistable *Wolbachia* dynamics in nature. However, other infections including *w*Mel in *D. melanogaster* cause minimal CI and persist despite incomplete maternal transmission. These infections may show Fisherian dynamics [12], [30], [31], consistent with the apparent global spread of alternative forms of *w*Mel, none of which causes appreciable CI [32].

Here we present *Wolbachia* and mtDNA data that document the sequential recent spread of two *Wolbachia* infections in eastern Australian *D. simulans* populations. Previous research [33] indicated that *D. simulans* were polymorphic for a novel *Wolbachia* infection (*w*Au) present at a low frequency that induced no detectable CI. The persistence of this infection is most easily understood if it increases fitness consistent with *F*(1−*μ*)>1. Although various potential fitness advantages have been associated with *Wolbachia* infections in the laboratory, including virus protection [34], [35] and fecundity increases [36], [37], they have not been demonstrated in nature for *w*Au or any other *Wolbachia* strain [38].

We show that the temporal and spatial data for *w*Au, and for *w*Ri that has recently invaded Australia [39], are best explained by postulating that each variant increases host fitness in nature. We also present a new observation relevant to the history of *w*Ri spread in Californian populations of *D. simulans*. These new data suggest that *w*Ri invasion may be consistent with a Fisherian rather than a bistable wave, increasing our understanding of *Wolbachia* infection dynamics in nature and supporting theoretical analyses [22] suggesting that the global distribution of *Wolbachia* throughout insects and other arthropods is most easily understood if these infections tend to spread deterministically from low initial frequencies.

## Results

### *Wolbachia* Infection Frequency Estimates

We assayed individual *D. simulans* samples for *Wolbachia* infection status and strain type using a real-time PCR/high resolution melt (RT/HTM) method designed to amplify a fragment of the *wsp* gene [39]. Samples which successfully amplified using this method yielded amplicons with melting temperature peak rates (*T_m_*) that clustered into two distinct groups which showed a consistent difference of ∼0.5°C. *Wolbachia* strain type for positively infected *D. simulans* individuals was assigned on this basis. Positive controls where DNA extracted from both a *w*Au-infected and a *w*Ri-infected individual fly was combined and amplified in the same reaction produced intermediate and less distinct *T_m_* peaks. This pattern was never observed for amplicons derived from individual specimens, suggesting that double infections are unlikely to occur amongst Australian field populations of *D. simulans* at any appreciable frequency. Sequencing of the amplicons derived from 13 of these samples using a standard PCR method with primers *wsp*_81F_Fwd: 5′-TGGTCCAATAAGTGATGAAGAAAC-3′ and *wsp*_691_Rev: 5′-AAAAATTAAACGCTACTCCA-3′ [40] confirmed that 9 samples from the high-*T_m_* cluster were the *w*Ri haplotype (cf. GI: 225591853), whilst the remaining 4 from the low-*T_m_* cluster were *w*Au (cf. GI: 2687519). For a further 5 high-*T_m_* and 5 low-*T_m_* cluster individuals we also obtained sequence data using standard PCR methods for each of the *Wolbachia* MLST genes *gatB*, *coxA*, *hcpA*, *ftsZ* and *fbpA* [41], and for the *sucB* gene [42]. These samples were drawn from isofemale lines derived from diverse geographic locations including, for the high-*Tm* cluster, lines established in 2011 from Brisbane, Melbourne and Gosford, New South Wales; and for the low-*T_m_* cluster, recently sourced lines from Perth and Geraldton (Western Australia), Y6 originally from Cameroon, West Africa (E.A. McGraw, *pers. comm*.), and Coff1 sourced from Coffs Harbour, New South Wales in the mid-1990s bearing the originally designated *w*Au infection [33]. A single haplotype was obtained for the high-*T_m_* samples which showed 100% sequence identity with the *w*Ri strain of *Wolbachia* (cf. GI:225629872). A single distinct haplotype was also obtained for the low-*T_m_* samples which was identical to previous data for the *w*Au strain at the *sucB* locus (cf. GI:84028372).

Further validation of these results was obtained using the RT/HTM method with two sets of strain-specific primers (*w*Ri_*wsp*_Fwd: 5′-TGATGTTGAAGGGCTTTATTCACAG-3′; *w*Ri_*wsp*_Rev: 5′-GTATCTGGGTTAAATGCTGCACCTG and *w*Au_*wsp*_Fwd: 5′-TGATGTTGAAGGAGTTTATTCATAC-3′; *w*Au_*wsp*_Rev: 5′-TTTGCTGGGTCAAATGTTACATCTT-3′). 34 of the original samples from the high-*T_m_* cluster each amplified successfully using the *w*Ri-specific primers but did not amplify with the *w*Au primers, whilst the reverse was true for 27 samples from the original low-*T_m_* cluster.

**Figure 1:**
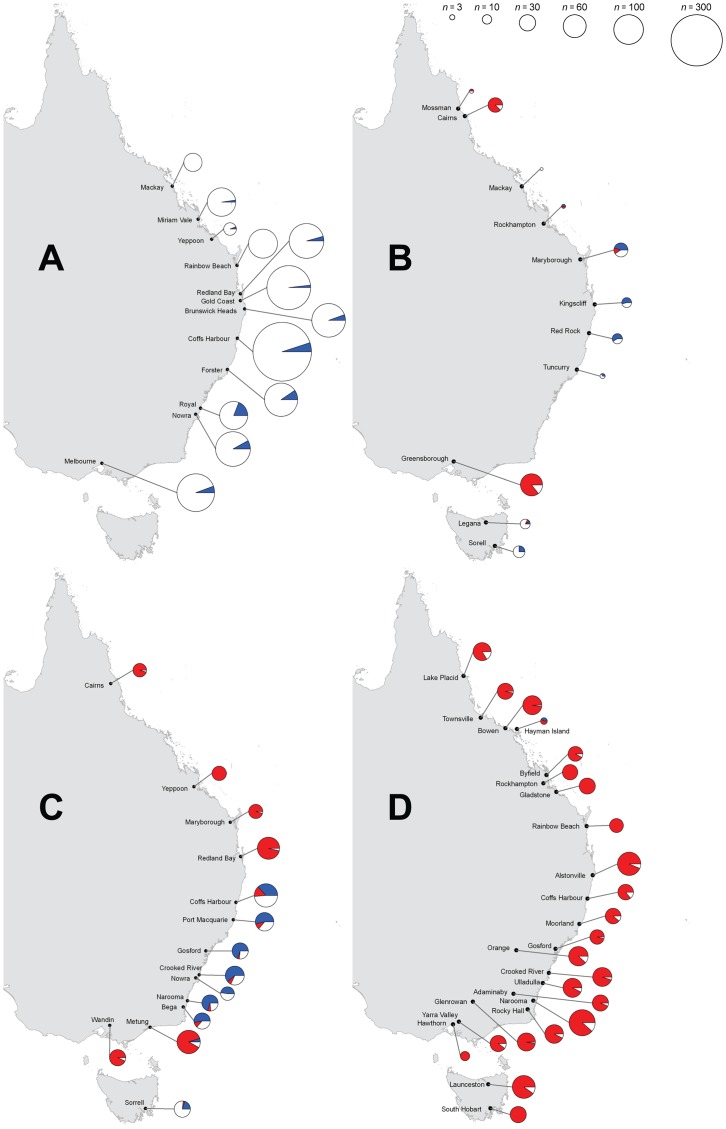
Location of *Drosophila simulans* collection sites in eastern Australia and the frequency of infection by *w*Au and *w*Ri in (A) 1994 (data adapted from Hoffmann et al. [33]), (B) 2004, (C) 2008 and (D) 2011/12. Blue shaded parts of the pie are *w*Au-infected *D. simulans*, red shaded are *w*Ri-infected and unshaded are uninfected individuals. Pie size represents sample size (*n*).

Results for samples collected in 2004 (*n* = 162), 2008 (*n* = 499), and 2011/12 (*n* = 799) are summarised in Figure 1 (full details in Table S1). In contrast to the infection frequencies found for samples collected in 1993/94 [33; Figure 1], a second *Wolbachia* strain (*w*Ri) was already prevalent amongst some east Australian *D. simulans* populations by 2004, both in the far north (Cairns) and the south (Melbourne); it occurred at a lower frequency further down the Queensland coast (Maryborough) and in northern Tasmania, but was not yet present in the central coastal area of northern New South Wales or in southern Tasmania. Additionally, for three central populations (Maryborough, Kingscliff and Red Rock), *w*Au infection frequencies increased significantly compared to geographically equivalent populations sampled previously (*P*<0.001, *G*-test). Ballard [43] also reports significantly higher *w*Au frequencies for samples collected in December 1999 from both Coffs Harbour and Brisbane than those previously found by Hoffmann *et al.* [33].

The 2008 data show *w*Ri spreading considerably since 2004, being at high frequency throughout Queensland, and now detected in both southern Tasmania and 6 of 7 populations from New South Wales. By 2011/12, *w*Ri was at high frequency at every location we sampled in mainland eastern Australia and Tasmania (*n* = 794) with an overall frequency of 0.929 (0.909, 0.946); whilst the *w*Au infection was only detected from an island (Hayman Island) ∼29 kms offshore from Airlie Beach in Queensland. We did not detect *w*Ri infection from two sites in Western Australia sampled in late 2011 and early 2012. Samples from Perth (*n* = 105) and Geraldton (*n* = 40), 370 km to the north, were polymorphic for the *w*Au infection, with infection frequencies of 0.648 (0.548, 0.738) and 0.500 (0.338, 0.662) respectively or 0.607 (0.522, 0.687) when pooled. Populations of *D. simulans* in Western Australia are separated from those in the east by ∼2000 km of dry habitat, preventing direct migration.

### CI Assay

Laboratory crosses indicate that the *w*Ri-infected Australian flies have a CI phenotype indistinguishable from California *w*Ri. Hatch rates (Table 1) from crosses between infected females from four north Queensland lines established from females collected in 2011 and five-day-old males from a *w*Ri-infected laboratory line provided no evidence for CI and were similar to the average hatch rate when females from these lines were crossed to males from an uninfected and a *w*Au-infected laboratory line. Females from these lines therefore behaved like *w*Ri lines in crossing type. In addition, when males from these lines were crossed, they were compatible with the *w*Ri line females, but generated incompatibility in crosses with females from both the uninfected and *w*Au-infected lines. All differences between reciprocal crosses were significant (Mann-Whitney U-tests) at the table wide α' = 0.05 level after corrections for multiple comparisons.

**Table 1:** Egg hatch rates (% hatch ± SE) from reciprocal crosses of females from four north Queensland (Nth Qld) lines with males from three laboratory lines (Riv88, w88, Coffs1) of previously determined infection status.

	Nth Qld ♀	Riv88 ♀	w88 ♀	Coffs1 ♀
	(*w*Ri)	(*w*Ri)	(Uninfected)	(*w*Au)
Riv88 ♂	92.6±2.29%	-	-	-
(*w*Ri)	(*n* = 15)			
w88 ♂	96.1±2.14%	-	-	-
(Uninfected)	(*n* = 16)			
Coffs1 ♂	90.3±6.3%	-	-	-
(*w*Au)	(*n* = 16)			
Nth Qld ♂	-	93.8±1.33%	5.2±2.22%	2.4±5.50%
(*w*Ri)		(*n* = 15)	(*n* = 18)	(*n* = 17)

### Maternal Transmission

To test maternal transmission of *w*Au in *D. simulans* in Western Australia, we established 55 isofemale lines from field-caught females obtained from two locations near Perth and from Geraldton in Western Australia in 2011/12. We assayed the *Wolbachia* infection status of the mothers and several F1 progeny, including between 10 and 12 progeny for each of 34 lines infected with *w*Au. From these lines, 350 progeny were tested, and all but 8 were found to be infected. This yields a field estimate for *μ* of 0.023 with 95% bootstrapped confidence interval (bCI) of 0.003, 0.049 based on 10,000 replicates). The bCI is broader than the 95% binomial confidence interval based on total numbers (0.01, 0.045), but is more appropriate because of heterogeneity in transmission frequencies across females [cf. 26].

We also tested maternal transmission of *w*Ri in eastern Australia using isofemale lines established from two locations near Melbourne in 2013. Ten F1 progeny were tested for each of 23 of these lines where the mother was found to be infected. All but 7 of the F1s were also infected, yielding a field estimate of *μ* of 0.026 (95% bCI 0.004, 0.057) which is consistent with previous field estimates of *μ* for *w*Ri in California [17], [26].

### Fecundity

**Figure 2:**
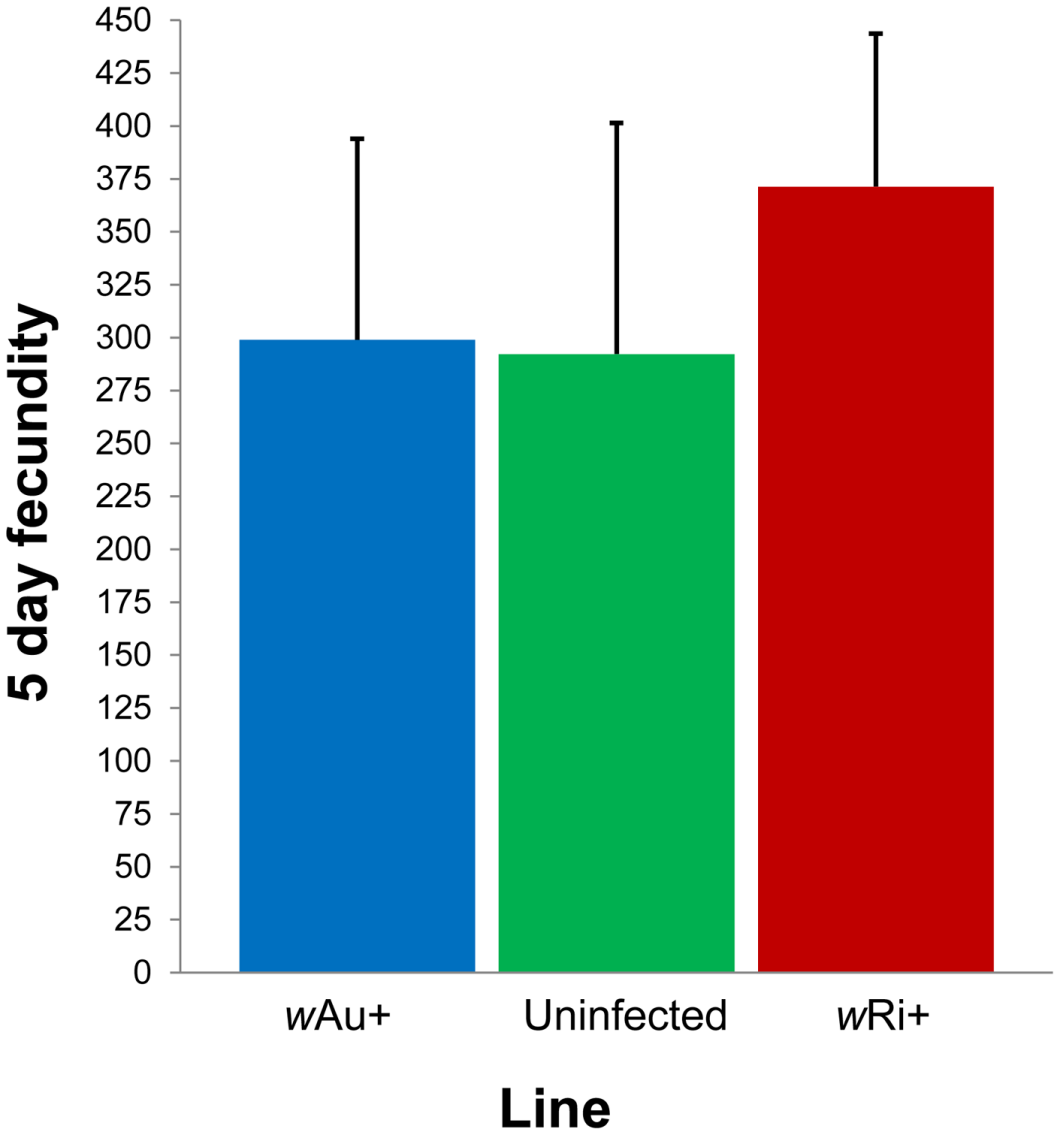
Fecundity assays on Australian *w*Ri-infected, *w*Au-infected, and uninfected laboratory lines of *D. simulans*. Mean number of eggs laid over 5 d by *w*Ri-infected (red bar), uninfected (green bar) and *w*Au-infected (blue bar) females. Error bars are standard errors.

The *w*Ri infection has previously been found associated with significant fecundity increases in *D. simulans* lines from California [37]. We performed a three-way comparison for fecundity using Australian *w*Ri-infected, *w*Au-infected and uninfected laboratory lines which had been outcrossed to a common genetic background for 5 generations. Results (Figure 2) indicate that females from the *w*Ri-infected line showed a marginally significant fecundity advantage relative to the uninfected line (*p* = 0.045, *t*-test) but strain differences were marginally non-significant in the three-way comparison (*F*_2,37_ = 2.7, *p* = 0.082).

### Mitochondrial Haplotypes

To examine the association between Australian *w*Au and *w*Ri infections across time, we obtained sequence data for a 1256 bp mtDNA fragment from 7 *w*Au-infected and 11 *w*Ri-infected samples, and found no variation apart from a single G to A base substitution previously identified by Ballard [43] (position 457 on our fragment, position 8201 in [43], see Table 2) with the former sequence (here denoted “A haplotype”) found only in *w*Au-positive samples and the latter (denoted “R haplotype”) only in *w*Ri-positive samples. Additional mtDNA haplotype sequence data obtained for 45 *Wolbachia* uninfected (*w*–) samples from 2004, 39 *w*– samples from 2008 and 6 *w*– samples from 2011 were similarly invariant, comprising only A or R haplotypes. The mtDNA haplotype for a further 9 *w*- samples from 2004, and 55 *w*- samples from 2008, was determined by treatment of PCR amplicons with the restriction enzyme *HinfI*. Full details of the mtDNA haplotypes determined for *w*- samples are summarised in Table S1. Both A and R haplotypes were detected amongst the 2004 and 2008 eastern Australian *w*- samples (simultaneously for some locations), whereas sequence data for *w*- samples collected in 2011 (*n* = 5) all confirmed the presence of the R haplotype. A single 2011 *w*– sample from Perth, Western Australia had the A haplotype.

**Table 2:** Polymorphism for mtDNA haplotypes.

Position*	DSR	AU23	DSW	CA61	Y54
1450	T	.	C	.	.
1560	C	.	.	T	.
8158	A	.	.	.	G
8201	A	G	G	G	.

The first three come from Ballard [43], the last two have not been previously described. The only positions shown are those at which the sequences differ from the DSR reference.

* Position descriptions follow those of [43].

To better understand the history of association of *w*Ri with *D. simulans*, we also examined mtDNA variation in California *D. simulans* stocks by sequencing two regions which included those identified in Table 3 of Ballard (2004) as showing the greatest variation among *si*II mtDNA haplotypes, positions 1175 to 1626 and 7838 to 8287. We examined mtDNA from a stock founded in 1961 in Nueva, California, ∼25 km SE of Riverside, the type locality for *w*Ri [28]. This stock is not infected with *Wolbachia*, and its origin pre-dates the initial 1985 *Wolbachia* analyses. It yielded a novel haplotype, denoted CA61, different from those described in Ballard (2004) by two base pairs from both the canonical “R haplotype,” which Ballard calls DSR, and the canonical haplotype (DSW) associated with northern California *D. simulans* prior to the invasion of *w*Ri. To better understand the novel CA61 haplotype, we examined mtDNA from two reference *w*Ri stocks, Riv84 and Riv88, collected in Riverside in 1984 and 1988, respectively, and from seven stocks (denoted Y26, Y35, Y36, Y42, Y46, Y54 and Y62) collected from an orchard approximately 5 km east of Winters, California in August 2011. All stocks apart from Y35 and Y62 were *w*Ri infected. The Y54 stock produced a novel haplotype which differs from the canonical DSR by one nucleotide (Table 1). As expected, the mtDNA from Riv84, Riv88, and all the other stocks matched DSR.

Given the paucity of sequence variation in *si*II *D. simulans* mtDNA sequences, and in the mtDNA associated with *w*Ri [43], [44], there is little statistical power to infer phylogenetic relationships among these sequences, apart from the fact that the sequences from the Indian Ocean seem to be part of a distinct clade (see Fig. 3 of [43]). However, with only one exception, all *w*Ri-infected stocks share the nucleotide A at position 8201, in contrast to the G found in DSW and CA61 (as well as AU23 and all other *w*Au-infected stocks examined by Ballard [43]. This substitution corresponds to the *HinfI* polymorphism described by Hale and Hoffmann [45] differentiating mtDNA of *w*Ri-infected stocks sampled worldwide from DSW. Nucleotide A was found at position 8201 in all *w*Ri-infected stocks examined worldwide (Ballard [43], 92 *w*Ri-infected stocks from California [46] and 29 of 30 additional *w*Ri-infected stocks [17]. The only exception may correspond to rare paternal transmission of mtDNA [cf. 47]. Given that uninfected flies from *w*Ri-infected populations quickly become associated with the mtDNA from their infected maternal ancestors [46], it seems unlikely that *w*Ri was present at an appreciable frequency near Riverside in 1961.

**Figure 3:**
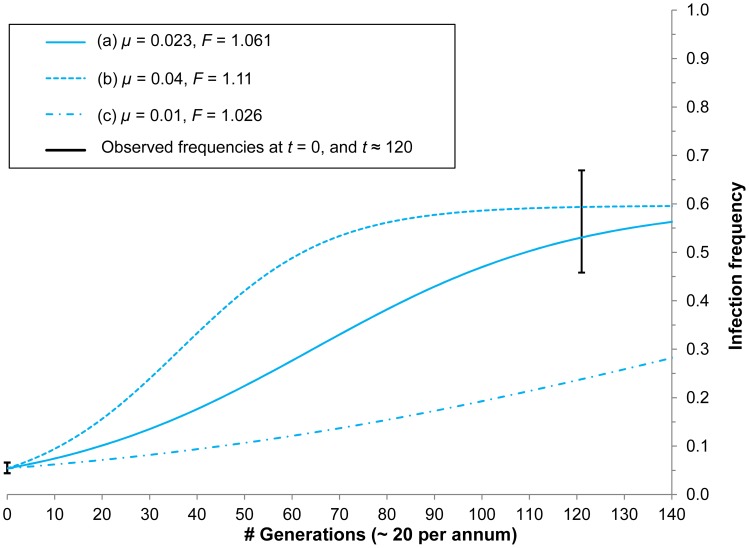
Predicted local *w*Au dynamics (where the infection increases host fitness (*F*_A_) and offsets occasional loss of infection through maternal leakage) showing the observed increase in infection frequency between 1993/4 and 1999 assuming ∼20 host generations per annum at subtropical latitudes. If the stable equilibrium frequency 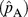 for *w*Au is ≈0.6, parameter combinations (a) and (b) are sufficient to explain the observed rate of increase, whereas combination (c) is not.

### Mathematical Inferences

As noted by Hoffmann and Turelli [12], for a *Wolbachia* variant such as *w*Au to persist despite imperfect maternal transmission and little CI, it must increase fitness sufficiently to offset imperfect transmission, *i.e.*, *F*(1−*μ*)>1. Let I_A_ (I_R_) denote individuals infected with *w*Au (*w*Ri), and let U denote uninfected individuals. We measure fitness of I_A_ and I_R_ females relative to uninfected females, and denote their relative fitnesses *F*_A_ and *F*_R_. Let *μ*_A_ (*μ*_R_) denote the frequency of U ova produced by I_A_ (I_R_) females. The condition for *w*Au to increase when rare is *F*_A_(1−*μ*_A_)>1. When this is satisfied, *w*Au will reach a stable equilibrium frequency of
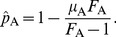
(1)The *w*Au frequencies observed in southern Queensland and northern New South Wales in 1999 and 2004, as well as the 2011/12 *w*Au frequencies in Western Australia, all suggest an equilibrium frequency for *w*Au of roughly 0.6. Using (1) and our estimate *μ*_A_≈0.023 implies *F*_A_≈1.061. Given the wide confidence interval for *μ*_A_ (0.003, 0.049), (1) implies that plausible values for *F*_A_ range from 1.008 to 1.140.

However, estimates of *F*_A_ are further constrained by the observed *w*Au frequency changes. A pooled frequency estimate (*n* = 1641) for mainland eastern Australia in 1993/1994 [33] is 0.054, with 95% confidence interval (0.044, 0.066); by December 1999, the estimated frequency (*n* = 92) had increased to 0.565 (0.458, 0.669). This increase occurred over about 120 generations, assuming roughly 20 generations per year for 6 years. With *F*_A_ = 1.061 and *μ*_A_ = 0.023, *p*_A_ would increase from 0.054 to 0.529 in 120 generations, consistent with our data. If *μ*_A_ were significantly smaller, say 0.01, (1) with 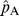 = 0.6 implies *F*_A_ = 1.026. Assuming *μ*_A_ = 0.01 and *F*_A_ = 1.026, *p*_A_ would increase in 120 generations from 0.054 to only 0.229, far below our 1999 estimate. However, if *μ*_A_ were appreciably larger, say 0.04, (1) with 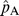 = 0.6 implies *F*_A_ = 1.11. With those values, *p*_A_ would increase from 0.054 to 0.597 in 120 generations. Thus, our frequency estimates and maternal transmission data are consistent with a positive fitness effect for *w*Au on the order of 5–10%, but inconsistent with appreciably smaller fitness advantages of 3% or less (Figure 3). Given the uncertainty of our estimates for both the apparent stable equilibrium frequency, 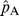, and the maternal transmission rate, *μ*_A_, for *w*Au, our data documenting the frequency increase of *w*Au from 1993 to 1999 provide the most robust estimate for the fitness advantage it seems to induce.

As shown in Materials and Methods, once *w*Au is established, *w*Ri will tend to increase when very rare only if
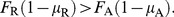
(2)The roughly simultaneous spread of *w*Ri from three geographically disparate foci in north Queensland, Victoria and Tasmania (Figure 1) indicates that condition (2) was met. Moreover, the observed temporal displacement of *w*Au by *w*Ri is consistent with (2) and the levels of CI and maternal transmission for *w*Ri observed in California [17], [26]. For instance, the *w*Ri and *w*Au infection frequencies estimated in March–May 2008 from seven coastal cities from New South Wales were statistically consistent (*G* test; *G* = 20.39, *P* = 0.06) with pooled frequencies (*n* = 253) of 0.09 and 0.54 for *w*Ri and *w*Au respectively (and 37% uninfected). In 2011, seven New South Wales coastal populations spanning the same range of latitudes were sampled (*n* = 302); again their infection frequencies were statistically consistent (*G*-test; *G* = 3.74, *P*>0.68), but *w*Au had been eliminated and the pooled frequency estimate for *w*Ri had risen to 0.91 (0.88, 0.94). We conjecture that there may be on the order of 15 generations per year on average in this area of coastal New South Wales (comparable to the Central Valley of California, versus 20 for sub-tropical coastal populations farther north).

**Figure 4:**
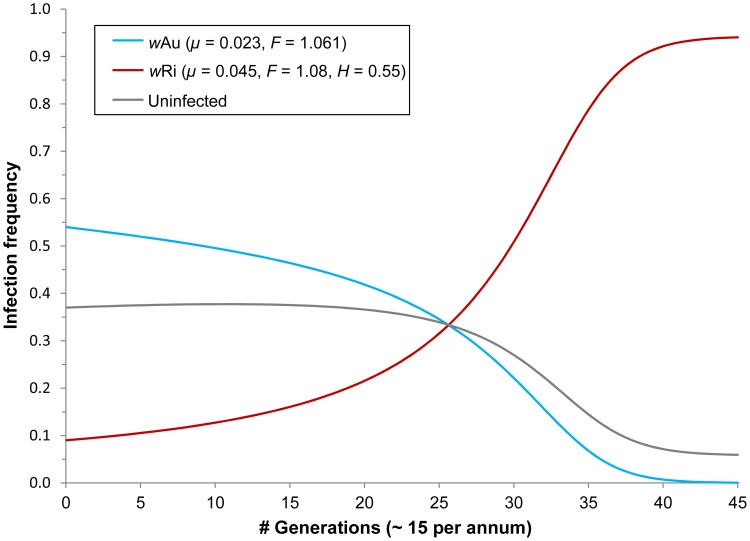
Predicted local dynamics for *w*Au displacement by *w*Ri to account for the speed of change observed for both infections along coastal New South Wales between 2008 and 2011 (assuming ∼15 host generations per annum for temperate latitudes). *F*_R_ (the fitness of *w*Ri-infected hosts relative to uninfecteds) is predicted to be on the order of 1.08 or higher in a simple model (employing previously estimated parameter values of *F*_A_ = 1.061 and *μ*_A_ = 0.023 for *w*Au, and *μ*_R_ = 0.045 and *H* = 0.55 for *w*Ri).

To compare these data to the predictions of our simple model (see Eqs. 3 below), we need estimates of fitness effects, maternal transmission rates and CI. As above, we assume *F*_A_ = 1.061 and *μ*_A_ = 0.023. Assuming that *H*, the relative hatch rate from incompatible fertilizations (*i.e.*, sperm from *w*Ri-infected males fertilizing either uninfected or *w*Au-infected ova), and the fidelity of maternal transmission were as observed in California, *e.g.*, *H* = 0.55 and *μ*_R_ = 0.045 [17], [26], we predict that starting with *p*_A_ = 0.54 and *p*_R_ = 0.09, *p*_A_ will fall below 0.01 within 40 generations only if *F*_R_ is on the order of 1.08 (Figure 4). After 45 generations, these parameter values imply that *p*_R_ should rise from 0.09 to very near its predicted stable equilibrium value of 0.94, consistent with our data.

The stable equilibrium frequency of *w*Ri is very insensitive to *F*_R_ [27]. For instance, with *H* = 0.55 and *μ*_R_ = 0.045, as *F*_R_ increases from 0.95 to 1.1, 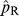 increases from 0.93 to only 0.94. In contrast, the unstable equilibrium for *w*Ri (when entering an uninfected population) plummets from 0.22 to 0, converting the predicted spatial dynamics from bistable to Fisherian. Our temporal data are too coarse to approximate accurately the speed of *w*Ri's spatial spread. However, between 2008 and 2011, *w*Ri spread southward from Coffs Harbour and northward from Bega (Figure 1), filling in roughly 1000 km of the New South Wales coast in three years. The speed of this bidirectional spread is comparable to the northward spread of *w*Ri observed in California, roughly 100 km/year [11]. Such rapid spatial spread can be explained by human-mediated, long-distance dispersal and Fisherian local dynamics that allow rare long-distance migrants to greatly accelerate spatial advance [25, Ch. 5].

## Discussion

Despite the ubiquity of *Wolbachia* in natural populations of arthropods, there are very few documented examples of *Wolbachia* spread [10], [11]. This has limited our understanding of *Wolbachia* spatial dynamics, with bistable spread for CI-causing *Wolbachia* suggested by imperfect maternal *Wolbachia* transmission by wild-caught females [15], [29] and demonstrated fecundity costs for infected females in the laboratory [17], [48], [49]. However, bistable dynamics cannot explain the persistence of *Wolbachia* that cause little or no reproductive manipulation [12]. Moreover, as emphasized by Fenton et al. [22], bistable dynamics are difficult to reconcile with molecular data indicating that the widespread occurrence of *Wolbachia* is attributable to rare horizontal transmission events involving one or very few infected founders [3], [44], [50], [51]. These phenomena are more easily explained by Fisherian dynamics [22], in which the frequency dynamics of rare *Wolbachia* infections are dominated by positive fitness effects. Our temporal and spatial data, describing the sequential spread of two *Wolbachia* strains (*w*Au and *w*Ri) through natural populations of *D. simulans* along the east coast of Australia over the last two decades, support the view that both strains may have spread under Fisherian dynamics.

The recent spread of *w*Ri is particularly compelling, with apparently independent introductions in both the north and south of the country between 1994 and 2004, leading to near-fixation of *w*Ri in all populations sampled on the east coast mainland of Australia by late 2011/early 2012. The appearance of *w*Ri in three geographically separated locations – Queensland, Victoria and Tasmania – in 2004 cannot be explained as a single bistable wave that originated and spread from elsewhere. In 1994, before the arrival of *w*Ri, *w*Au was rare in several populations. Ballard's (2000) data from 1999 and our samples from 2004 and 2008 (Figure 1) document *w*Au frequency increases across the Australian east coast. Given that *w*Au does not induce CI [33], its spread from low frequency in multiple populations implies that it confers a fitness benefit. The rapid displacement of *w*Au by *w*Ri suggests that *w*Ri must enhance fitness even more than *w*Au.

Both *w*Au and *w*Ri have been detected in *D. simulans* populations around the world [43], [52], [53], and these infections have co-occurred previously (e.g. Sangoqui and Rocafuerta, Ecuador in 2000; Brooksville, FL, USA in 2002 [43], and possibly also Japan [17]). The *w*Au infection in *D. simulans* may have a Neotropical origin as a result of a relatively recent horizontal transfer event from *Drosophila willistoni* [54]. The origin of *w*Ri in *D. simulans* is less certain, but very closely related *Wolbachia* have been found in various *Drosophila* that co-occur with this cosmopolitan species [e.g.], [ 54], [55,56]. It is not known how either strain was introduced into Australian *D. simulans* populations, but our data suggest that both introductions occurred within the last few decades. The pooled frequency of *w*Ri (92.7%, Table S1) in our 2011/12 eastern Australian samples (including Tasmania) is consistent with the stable equilibrium frequency found in California over the past two decades [∼93%]; [ 17], [26,37]. This equilibrium frequency is determined primarily by significant CI and imperfect maternal transmission. Our data from crossing experiments, and mtDNA and *Wolbachia* sequencing suggest that the *w*Ri strain now found throughout eastern Australia is essentially identical to that initially identified in California [28] and subsequently found in Africa, Asia, Europe and South America [17], [43]. Genome comparisons support this (M. Turelli, unpublished data). Our mtDNA data from uninfected individuals in 2011/12 indicate that the R haplotype is being swept to fixation in eastern Australia as a consequence of the spread (with imperfect maternal transmission) of *w*Ri [cf. 46]. A previous mitochondrial sweep of the A haplotype associated with *w*Au may have also occurred, but the ancestral haplotypes in eastern Australian *D. simulans* are not known.

*Wolbachia* and mtDNA are expected to be co-inherited maternally. Even though *Wolbachia* exhibit imperfect maternal transmission (*μ*>0), mtDNA haplotypes associated with a given infection will be driven to fixation, by CI or other *Wolbachia*-associated positive fitness effects, if all offspring of infected females inherit maternal mtDNA [46]. Eventually, all individuals in the population have infected maternal ancestors who passed on their mtDNA even if not their *Wolbachia*. Although rare paternal transmission of *Wolbachia* has been detected in laboratory *D. simulans* [48], [49], Turelli *et al.* [46] and Turelli & Hoffmann [17] inferred that paternal transmission or horizontal transfer must be rare in nature. Similarly, there is evidence for rare paternal inheritance of mtDNA in *D. simulans* [47], [57], [58], but it is apparently too rare to create incongruity between *Wolbachia* and mtDNA lineages [43]. *Drosophila simulans* populations worldwide carry three distinct mtDNA haplotype groups, with very little intra-haplogroup variation [44], [59], [60]. Ballard [43] found both *w*Au and *w*Ri only in flies carrying *si*II haplogroup mtDNA. He identified a single (synonymous) G to A base substitution (see Table 2) that was consistently different between *w*Au-infected versus *w*Ri-infected flies respectively, and matched a restriction enzyme polymorphism identified by Hale and Hoffmann [45]. In our analyses, all *w*Ri flies had the same base substitution as identified by these researchers, consistent with the hypothesis of a unique origin for *w*Ri in *D. simulans*.

Turelli and Hoffmann [11], [17] had previously suggested bistable spatial dynamics of *w*Ri in California populations of *D. simulans*. However, the rate at which *w*Ri spread spatially, on the order of 100 km per year, is more consistent with Fisherian dynamics whose deterministic spread from very low frequencies allows rare long-distance dispersal to greatly accelerate spatial advance. Assuming that the mtDNA haplotype of the 1961 strain we characterized was widespread in southern California, our new analysis suggests that *w*Ri was not yet at appreciable frequency at this time, and that the invasion detected by Turelli and Hoffmann [11] may reflect a rapid spatial spread from populations farther south.

The speed of *w*Ri spread throughout eastern Australia is comparable to the spread of *w*Ri in California, on the order of 100 km/year [11]. The infection was common in two populations sampled in 2004 (Melbourne and Cairns), then swept to near fixation in all east Australian mainland populations and Tasmania by 2011/12. Although some genes and traits exhibit clinal variation over this range [61], [62], several molecular markers indicate that *D. simulans* is effectively a single panmictic unit throughout eastern Australia, including Tasmania [63]. Thus, there are no apparent barriers to dispersal that would isolate populations from the spread of *w*Ri. We did not, however, detect *w*Ri in Western Australia, with the *w*Au infection persisting at relatively high frequency (≈60%). Western Australian populations of *D. simulans* may be geographically isolated from eastern Australian populations, although once *w*Ri is introduced it is likely to sweep through this area in the coming decades.

The Drosophila-Wolbachia dynamics have been used to interpret and model the spread of *Wolbachia*-infected *Aedes aegypti* mosquitoes in Australia [7]. In contrast to the apparent Fisherian spatial dynamics of *w*Au and *w*Ri, these releases seem to follow bistable dynamics, with strong evidence for a non-trivial unstable equilibrium frequency, on the order of 10–20% [6], [7]. After *w*Mel was driven to a high frequency in two relatively isolated populations by 10 weeks of releases, it increased to near-fixation, and has remained at over 90% frequency for the past 2 years [7; unpublished data]. Although the infection was detected in disjunct residential areas near the release sites soon after the initial releases, it has not persisted or spread in these areas [7; unpublished data], as would be expected under Fisherian dynamics. Given the evolutionary pressure for *Wolbachia* to evolve towards mutualism with its hosts [37], [64], a decreasing unstable point may alter these dynamics in the future [13] particularly if fitness costs produced in novel hosts are overcome.

In summary, our results suggest that the *w*Au and *w*Ri *Wolbachia* infections of *D. simulans* spread at least part of the time in a Fisherian manner. The *w*Au infection increased in frequency despite having imperfect transmission and not causing CI. Its spread and apparent equilibrium make sense only if it increased host fitness. Over the past decade, *w*Au was rapidly displaced by *w*Ri throughout eastern Australia. The rapidity of this replacement starting from three locations in 2004 indicates that *w*Ri also increases host fitness in nature, at least under some circumstances. These data plus evidence that *w*Ri entered southern California after 1960 lead us to reinterpret that canonical example of *Wolbachia* spread in California as Fisherian rather than bistable. Depending on the rapidity with which *Wolbachia* adapt to novel hosts, its spread after artificial introductions to target species may be greatly accelerated.

## Materials and Methods

### Field Collections

Field-caught *D. simulans* samples were collected from various locations along the east coast of Australia including Tasmania during February–March 2004 [61] and March–May 2008 [see 39]. Further samples were collected from mainland eastern Australia at similar coastal and some inland localities, and from the Perth region of Western Australia during 2011, and from both Geraldton (Western Australia) and Tasmania in early 2012. All samples were preserved in 100% ethanol and stored at −20°C. Specimens subsequently used in PCR analysis were males except where a few isofemale lines had been established prior to preservation of the mother. Species identity was established by male genital arch morphology and confirmed by molecular methods [39].

The new California samples were from isofemale lines (Y26, Y35, Y36, Y42, Y46, Y54 and Y62) established in August 2011. The collection site was a peach orchard approximately 5 km east of Winters, CA. The stocks were maintained as isofemale lines until analysed. The 1961 Nueva, California stock was obtained from the Drosophila Species Stock Center at University of California, San Diego (stock #14021-0251.006).

### *Wolbachia* Infection Status

DNA extractions were performed using a standard Chelex based method, and assays for *Wolbachia* infection status and strain type were performed with a RT/HRM method using the Roche LightCycler® 480 system as previously described [39]. Briefly, a conserved set of primers (*wsp*_validation_Fwd: 5′-TTGGTTACAAAATGGACGACATCAG-3′ and *wsp*_validation_Rev: 5′-CGAAATAACGAGCTCCAGCATAAAG-3′) were used to target a variable ∼340 bp region of the *wsp* gene. The *w*Ri and *w*Au alleles are expected to differ by 22 SNPs and a single 3 bp indel over this *wsp* region, potentially yielding a significant difference in observed *T_m_* for the respective PCR amplicons, with the *w*Ri allele expected to have the higher average *T_m_* [39].

Samples from *D. simulans* isofemale lines previously identified as *w*Ri- or *w*Au-infected respectively, were included on each PCR plate as *Wolbachia* strain-type positive controls. Further positive controls entailed equal volumes of DNA extracted from an individual fly of each type being combined in a single reaction. Separate *D. simulans* specific primers (*Dsim*_*RpS6*_Fwd: 5′-CCAGATCGCTTCCAAGGAGGCTGCT-3′; *Dsim*_*RpS6*_Rev: 5′-GCCTCCTCGCGCTTGGCCTTAGAT-3′) were used as a host species-specific control for each sample.

The RT-PCR/HRM conditions were as follows: 10 minutes at 95°C; 45 cycles of 10 seconds at 95°C, 15 seconds at 58°C, 30 seconds at 72°C; 1 minute at 95°C; 1 minute at 40°C. High resolution melting; temperature ramped up to 95°C at 0.02°/second with continuous fluorescence acquisition; 30 seconds at 40°C.

Individual samples were scored as *Wolbachia* infected, uninfected or inconclusive based on machine reported *wsp* and *RpS6* crossing point (Cp) relative and absolute value criteria and examination of individual *T_m_* profiles for evidence of non-specific products. A relatively small number of samples, which initially yielded inconclusive results, were retested.

Additional standard PCR amplification of *wsp*, *gatB*, *coxA*, *hcpA*, *ftsZ*, *fbpA* and *sucB* gene fragments for selected individual samples followed [41] with annealing temperatures of 54°C for *gatB*, *coxA*, *hcpA*, *ftsZ* and *sucB*, and 59°C for *wsp* and *fbpA*. PCR products were checked using a 2% agarose gel for presence of an unambiguous single band of expected size. Amplified DNA for selected samples which yielded a clear and positive PCR result were sent to Macrogen (Korea) for purification and sequencing. Sequence data obtained was analyzed using Geneious v6.1 (Biomatters, Auckland, NZ).

### mtDNA Analyses

Assays for mitochondrial DNA haplotype for a total of 172 individual Australian samples were performed using primers (*Dsim*_*si*II_7765_Fwd: 5′-ATTTAATATTCAAGCAATAGC-3′; *Dsim*_*si*II_8981_Rev: 5′-TTCTGGTTCTATAATTTTAGC-3′) designed to target a 1256 bp region of the *D. simulans* mitochondrial genome previously identified as containing at least one (synonymous) G to A base substitution at position 457 (outer strand) that was a characteristic difference between the haplotypes found to be associated with either the *w*Au or *w*Ri strains of *Wolbachia*, respectively [43].

Amplification of mtDNA was performed using a standard PCR method with the following conditions: 5 minutes at 94°C; 38 cycles of 30 seconds at 94°C, 1 minute at 55°C, 1 minute at 72°C; 5 minutes at 72°C; hold at 4°C.

PCR products were checked using a 2% agarose gel for the presence of an unambiguous single band of expected size. Amplified DNA for 108 samples that yielded a clear and positive PCR result were sent to Macrogen (Korea) for purification and sequencing. Sequence data obtained was inspected using Sequencher v4.5 (Gene Codes, Ann Arbor, Mi). Subsequently PCR amplicons for a further 64 samples were checked on a 2% agarose gel after digestion with the restriction enzyme *HinfI* [45].

For each California line, DNA was extracted from 30 flies as described in [65]. Following the position descriptions in [43], two sets of primers were used to amplify two mtDNA regions from positions 1034 to 1718 and from 7797 to 8425. The second region contains the G to A base substitution at position 8201 that distinguished the R haplotype from the A haplotype, associated with *w*Ri and *w*Au infection, respectively. (The primers used were: region 1: DSRmt1033+: 5′-CCAAAATGACTTGTAATCCA-3′ and DSRmt1719−: 5′-GCACCTAATATTAAAGGCACT-3′, region 2: DSRmt7796+: 5′-GCTACATCTCCAATTCGATTA-3′ and DSRmt8426−: 5′-TTTATATTCTTTTAGACAACATGG-3′.)

The PCR conditions were: 3 minutes at 94°C; 34 cycles of 30 seconds at 94°C, 30 seconds at 55°C, 75 seconds at 75°C; 8 minutes at 72°C; hold at 10°C. The PCR products were checked on a 2% agarose gel for an unambiguous band of the correct size. The PCR products were purified using the QIAquick PCR purification kit. The amplified fragments were Sanger sequenced by the UC Davis College of Biological Sciences UCDNA Sequencing Facility, using the same primers used for the PCR reaction. The sequencing results were analysed using ApE alignment tool (A plasmid Editor v2.0.45, by M. Wayne Davis).

To confirm the sequence coordinates we realigned the mtDNA genomes described by Ballard in [43], [44], [66] also including the *D. yakuba* mtDNA reference genome [67], and confirmed the positions of polymorphisms described in Table 3 of [43]. We then aligned our new sequences against this reference.

### CI Assay

Three separate laboratory lines previously confirmed to be *w*Ri-infected (Riv88), *w*Au-infected (Coffs 1) and uninfected (W88) respectively were used in reciprocal crosses with each of four separate lines (CBQ40, CBQ46, CBQ72 and CBQ80) established from field caught females collected in 2011 from north Queensland to test for CI. The level of CI was determined by mating virgin 5 d-old males to virgin females (5–8 d old). Males were mated once, and females were placed after mating in a vial with a spoon containing 5 ml of agar-treacle-yeast medium and left for 24 h at 25°C. The number of unhatched eggs was counted 24–32 h later. CI data (egg hatch rates) were angular transformed prior to analysis. Mann-Whitney U-tests were used to compare CI levels between the different lines. Multiple comparisons were corrected at the table wide α' = 0.05 level using the Dunn-Sidak method [68].

### Fecundity

Separate mass bred *w*Au-infected and uninfected fly lines were established from 20 to 30 Western Australian isofemales lines for which infection status had previously been determined. Virgin females from each of these mass bred lines were then mated with a similar number of field caught Brisbane males which had been aged in the laboratory for at least 5 d to reduce the impact of CI. An equivalent mass bred *w*Ri-infected line was established from virgin female F1 progeny of field collected Brisbane females mated with males from the uninfected mass bred line. Each mass bred line was then outcrossed to field collected aged Brisbane males which were also aged for at least 5 d for a further 4 generations to homogenise the genetic backgrounds. Lines were then retested to confirm infection status. Flies were reared at low densities by transferring 25 eggs into vials on ∼20 ml of agar-cornmeal-yeast medium. Pairs of virgin females and males were transferred to fresh medium vials for 2 d, then females were transferred to vials with spoons containing 5 ml of medium with 20% fresh live yeast paste added to the medium surface. Spoons were replaced every 24 h for 5 d and eggs counted. Twelve to 13 females were assayed for each line. Model I ANOVA (analysis of variance) and *t*-tests were used to compare fecundity between lines.

### Mathematical Analyses

We address two issues: first, the conditions for the initial spread and maintenance of *w*Au in an isolated population, then the conditions for its displacement by *w*Ri. Let I_A_ (I_R_) denote an individual infected with *w*Au (*w*Ri), and let U denote an uninfected individual. Assuming discrete generations, we denote the frequency of these three types of adults in generation *t* by *p*_A,*t*_, *p*_R,*t*_ and *p*_Ø,*t*_. No individuals doubly infected for *w*Au and *w*Ri have been found (and each infection is associated with distinct mtDNA haplotypes, [43]), hence we assume *p*_A,*t*_+*p*_R,*t*_+*p*_Ø,*t*_ = 1. We measure fitness of I_A_ and I_R_ females relative to uninfected females, and denote their relative fitnesses *F*_A_ and *F*_R_. In principle, these *Wolbachia* may affect viability or fecundity. Given that our data indicate relatively small fitness effects (see Results), we can simplify the analysis by approximating fitness differences as female fecundity variation with no viability effects.

Field-collected I_A_ and I_R_ females have both been shown to produce uninfected offspring. Let *μ*_A_ (*μ*_R_) denote the frequency of U ova produced by I_A_ (I_R_) females. Before *w*Ri arrives in the population, *p*_A,*t*_+*p*_Ø,*t*_ = 1; so we need only keep track of *p*_A,t_. Because *w*Au causes no CI,
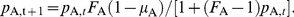
(3)Assuming *F*_A_(1−*μ*_A_)>1, this produces the stable equilibrium (1) in Results. (Equilibrium (1) is equivalent to the standard mutation-selection equilibrium for haploids, in which the uninfected, less-fit type is maintained at frequency *μ*/*s*, where *μ* = *μ*_A_ is the fraction of uninfected ova produced by infected mothers and 1−*s* = 1/*F*_A_ is the relative fitness of U females.) Equilibrium (1) implies that if 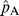 and *μ*_A_<<1 are known, *F*_A_≈1+*μ*_A_/(1−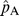).

To understand the dynamics of *w*Ri entering a population with *w*Au, note that both Hoffmann et al. [33], and James and Ballard [53] demonstrated that *w*Au infection is not able to rescue CI caused by *w*Ri. Indeed, *w*Ri induces the same level of CI in matings with both uninfected (U) and *w*Au-infected (I_A_) females. The joint dynamics of *p*_A,*t*_, *p*_R,*t*_ and *p*_Ø,*t*_ follow a simplified version of the recursions for double and single infections derived by Hoffmann and Turelli [12, Eqs. 2.5]. Let *H* = 1−*s*_h_<1 denote the relative hatch rate of embryos produced by I_A_ or U ova fertilized by sperm from I_R_ males. The relevant recursions are
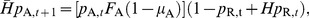
(4a)

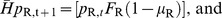
(4b)

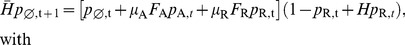
(4c)

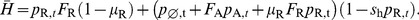
(4d)Note that 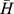 in (4d) is just the sum of the right hand sides of (4a–c). Each of these three expressions has a simple interpretation as the product of two terms, the first (in square brackets) proportional to the fraction of ova of each type, the second proportional to the fraction of fertilized ova that hatch. (In (4b) this second term is one, because *w*Ri-infected ova are compatible with all sperm.) Given that the three frequencies sum to 1, there are only two independent variables.

Now consider the conditions for *w*Ri to increase when it is extremely rare and *w*Au is at equilibrium with U, as described by Eq. (1). When *p*_R,*t*_≈0, 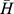
≈*p*_Ø,*t*_+*F*_A_ *p*_A,*t*_. When *w*Au and U are at equilibrium, Eq. (3) implies that *p*_Ø,*t*_+*F*_A_ *p*_A,*t*_ = *F*_A_(1−*μ*_A_). Hence, from (4b), we see that the condition for *w*Ri to increase when very rare is condition (2) provided in Results. This is just the condition for spread of a *Wolbachia* variant that is completely compatible with the existing type [64]. Because essentially no CI occurs when *w*Ri is very rare, the incompatibility of *w*Ri with *w*Au does not enter condition (2).

When (2) is not met, *p*_R,*t*_ increases only once it exceeds a frequency threshold so that the fitness advantage I_R_ individuals receive from CI overcomes their frequency-independent disadvantage relative to variant A. This condition is a simple generalization of the unstable equilibrium that arises for a single *Wolbachia* infection that satisfies *H*<*F*(1−*μ*)<1.

### Accession Numbers

Sequences for the MLST genes *gatB*, *coxA*, *hcpA*, *ftsZ* and *fbpA*, and the *sucB* gene obtained in this study have been deposited in GenBank (http://www.ncbi.nlm.nih.gov/Genbank) under accession numbers KF278668–KF278673.

## Supporting Information

Table S1 *Wolbachia* infection frequencies and mtDNA haplotypes by location.
(DOC)
