# Supplementary material for: Rapid Sequential Spread of Two Wolbachia Variants in Drosophila simulans
Source: PLoS Pathog. 2013 Sep 12;9(9):e1003607. doi: 10.1371/journal.ppat.1003607 (PMC3771877; doi:10.1371/journal.ppat.1003607)
Supplement: Table S1 — Wolbachia infection frequencies and mtDNA haplotypes by location. (DOC) [file ppat.1003607.s001.doc]

| **Eastern Australia** | | | | | | | | | |
| --- | --- | --- | --- | --- | --- | --- | --- | --- | --- |
| Locality | Collection date | Latitude | Longitude | *n* | *w*Au+ | *w*Ri+ | Uninf | ‘R’ type^1^ | ‘A’ type^2^ |
| Mossman, Queensland | June, 2004 | -16.457° | 145.373° | 2 | 0 | 1 | 1 | 1 | 0 |
| Cairns, Queensland | June, 2004 | -16.914° | 145.762° | 24 | 0 | 21 | 3 | 3 | 0 |
| Mackay, Queensland | June, 2004 | -21.147° | 149.185° | 1 | 0 | 0 | 1 | 1 | 0 |
| Rockhampton, Queensland | June, 2004 | -23.401° | 150.491° | 2 | 1 | 1 | 0 | - | - |
| Maryborough, Queensland | April / June, 2004 | -25.546° | 152.685° | 22 | 10 | 3 | 9 | 1 | 7 |
| Kingscliff, New South Wales | March, 2004 | -28.257° | 153.576° | 11 | 6 | 0 | 5 | 0 | 5 |
| Red Rock, New South Wales | March, 2004 | -29.983° | 153.229° | 12 | 7 | 0 | 5 | 0 | 5 |
| Tuncurry, New South Wales | March, 2004 | -32.170° | 152.498° | 3 | 1 | 0 | 2 | 0 | 2 |
| Greensborough, Victoria | April, 2004 | -37.700° | 145.101° | 56 | 0 | 48 | 8 | 8 | 0 |
| Legana, Tasmania | March, 2004 | -41.365° | 147.041° | 13 | 1 | 3 | 9 | 5 | 4 |
| Sorell, Tasmania | March, 2004 | -42.770° | 147.576° | 16 | 4 | 0 | 12 | 0 | 12 |
| 2004 totals: | | | | 162 | 30 | 77 | 55 | 19 | 35 |
|  | | | | | | | | | |
| Cairns, Queensland | March - May, 2008 | -16.923° | 145.774° | 21 | 0 | 20 | 1 | - | - |
| Yeppoon, Queensland | March - May, 2008 | -23.128° | 150.745° | 24 | 0 | 24 | 0 | - | - |
| Maryborough, Queensland | March - May, 2008 | -25.537° | 152.702° | 23 | 0 | 22 | 1 | 1 | 0 |
| Redland Bay, Queensland | March - May, 2008 | -27.612° | 153.302° | 56 | 0 | 54 | 2 | 1 | 0 |
| Coffs Harbour, New South Wales | March - May, 2008 | -30.296° | 153.116° | 62 | 23 | 9 | 30 | 2 | 9 |
| Port Macquarie, New South Wales | March - May, 2008 | -31.431° | 152.908° | 39 | 21 | 4 | 14 | 1 | 6 |
| Gosford, New South Wales | March - May, 2008 | -33.425° | 151.342° | 30 | 21 | 1 | 8 | 1 | 5 |
| Crooked River, New South Wales | March - May, 2008 | -34.738° | 150.806° | 41 | 25 | 3 | 13 | 3 | 8 |
| Nowra, New South Wales | March - May, 2008 | -34.913° | 150.603° | 21 | 10 | 0 | 11 | 2 | 9 |
| Narooma, New South Wales | March - May, 2008 | -36.287° | 150.108° | 30 | 21 | 2 | 7 | 0 | 7 |
| Bega, New South Wales | March - May, 2008 | -36.674° | 149.845° | 30 | 16 | 3 | 11 | 1 | 10 |
| Wandin, Victoria | March - May, 2008 | -37.779° | 145.428° | 30 | 0 | 28 | 2 | 2 | 0 |
| Metung, Victoria | March - May, 2008 | -37.892° | 147.853° | 62 | 3 | 54 | 5 | 5 | 0 |
| Sorrell, Tasmania | March - May, 2008 | -42.770° | 147.576° | 30 | 6 | 1 | 23 | 5 | 16 |
| 2008 totals: | | | | 499 | 146 | 225 | 128 | 24 | 70 |
|  | | | | | | | | | |
| Lake Placid, Queensland | October, 2011 | -16.871° | 145.674° | 37 | 0 | 31 | 6 | 2 | 0 |
| Townsville, Queensland | October, 2011 | -19.376° | 146.705° | 29 | 0 | 28 | 1 | 1 | - |
| Bowen, Queensland | October, 2011 | -20.016° | 148.195° | 42 | 0 | 41 | 1 | - | - |
| Hayman Island, Queensland | July, 2012 | -20.057° | 148.884° | 5 | 2 | 3 | 0 | - | - |
| Byfield, Queensland | October, 2011 | -22.824° | 150.664° | 25 | 0 | 23 | 2 | - | - |
| Rockhampton, Queensland | October, 2011 | -23.331° | 150.476° | 27 | 0 | 27 | 0 | - | - |
| Gladstone, Queensland | October, 2011 | -23.849° | 151.263° | 30 | 0 | 30 | 0 | - | - |
| Rainbow Beach, Queensland | October, 2011 | -25.906° | 153.091° | 22 | 0 | 22 | 0 | - | - |
| Alstonville, New South Wales | October, 2011 | -28.853° | 153.455° | 62 | 0 | 58 | 4 | 2 | - |
| Coffs Harbour, New South Wales | October, 2011 | -30.277° | 153.133° | 28 | 0 | 24 | 4 | - | - |
| Moorland, New South Wales | April, 2011 | -31.789° | 152.652° | 28 | 0 | 25 | 3 | - | - |
| Gosford, New South Wales | April, 2011 | -33.289° | 151.215° | 24 | 0 | 23 | 1 | - | - |
| Orange, New South Wales | April, 2011 | -33.373° | 148.855° | 43 | 0 | 38 | 5 | - | - |
| Crooked River, New South Wales | April, 2011 | -34.744° | 150.808° | 41 | 0 | 39 | 2 | - | - |
| Ulladulla, New South Wales | April, 2011 | -35.352° | 150.444° | 41 | 0 | 38 | 3 | - | - |
| Adaminaby, New South Wales | April, 2011 | -35.994° | 148.678° | 30 | 0 | 28 | 2 | - | - |
| Narooma, New South Wales | April, 2011 | -36.413° | 149.867° | 78 | 0 | 69 | 9 | - | - |
| Glenrowan, Victoria | April, 2011 | -36.470° | 146.235° | 37 | 0 | 36 | 1 | - | - |
| Rocky Hall, New South Wales | April, 2011 | -36.939° | 149.535° | 40 | 0 | 37 | 3 | - | - |
| Yarra Valley, Victoria | March, 2011 | -37.677° | 145.387° | 30 | 0 | 27 | 3 | - | - |
| Hawthorn, Victoria | November, 2011 | -37.833° | 145.035° | 10 | 0 | 10 | 0 | - | - |
| Launceston, Tasmania | January, 2012 | -41.441° | 147.156° | 60 | 0 | 54 | 6 | - | - |
| South Hobart, Tasmania | February, 2012 | -42.897° | 147.292° | 30 | 0 | 30 | 0 | - | - |
| 2011/12 total: | | | | 799 | 2 | 741 | 56 | 5 | 0 |
| **Western Australia** | | | | | | | | |  |
| Perth Hills, Western Australia | October, 2011 | -32.047° | 116.139° | 25 | 17 | 0 | 8 | 0 | 1 |
| South Perth, Western Australia | February, 2012 | -31.976° | 115.870° | 80 | 51 | 0 | 29 | - | - |
| Geraldton, Western Australia | April, 2012 | -28.767° | 114.620° | 40 | 20 | 0 | 20 | - | - |
| 2011/12 total: | | | | 145 | 88 | 0 | 57 | 0 | 1 |

^1^ Number of uninfected individuals determined to have the R mtDNA haplotype

^2^ Number of uninfected individuals determined to have the A mtDNA haplotype
